# Perspectives of Singaporean biomedical researchers and research support staff on actual and ideal IRB review functions and characteristics: A quantitative analysis

**DOI:** 10.1371/journal.pone.0241783

**Published:** 2020-12-31

**Authors:** Markus K. Labude, Liang Shen, Yujia Zhu, G. Owen Schaefer, Catherine Ong, Vicki Xafis

**Affiliations:** 1 Centre for Biomedical Ethics, Yong Loo Lin School of Medicine, National University of Singapore, Singapore, Singapore; 2 Biostatistics Unit, Yong Loo Lin School of Medicine, National University of Singapore, Singapore, Singapore; 3 Department of Medicine, Yong Loo Lin School of Medicine, National University of Singapore, Singapore, Singapore; 4 Division of Infectious Diseases, University Medicine Cluster, National University Health System, Singapore, Singapore; Rowan University School of Osteopathic Medicine, UNITED STATES

## Abstract

**Background:**

Biomedical research is overseen by numerous Institutional Review Boards (IRBs) in Singapore but there has been no research that examines how the research review process is perceived by the local research community nor is there any systematic data on perceptions regarding the review process or other research ethics processes and IRB characteristics. The aim of this study was to ascertain general views regarding the overall perceived value of ethics review processes; to measure perceptions about local IRB functions and characteristics; to identify IRB functions and characteristics viewed as important; and to compare these views with those of other international studies.

**Methods:**

An online survey was used with the main component being the IRB-Researcher Assessment Tool (IRB-RAT), a validated tool, to evaluate perceptions of *ideal* and *actual* IRB functions and characteristics held by Singaporean researchers and research support staff. Data were analysed descriptively first, with mean and SD of each item of IRB-RAT questionnaire reported, excluding the respondents whose answers were *unknown* or *not applicable*. The Wilcoxon Sign Rank test was used to compare the ideal and actual ratings of each IRB-RAT item, while the Mann-Whitney U test was used to compare the ratings of each IRB-RAT item between respondents with different characteristics. The Z-test was used to compare the mean ratings of our cohort with the mean ratings reported in the literature. The correlation between our mean ideal scores and those of two international studies also employing the IRB-RAT was examined.

**Results:**

Seventy-one respondents completed the survey. This cohort generally held positive views of the impact of the ethics review process on: the quality of research; establishing and maintaining public trust in research; the protection of research participants; and on the scientific validity of research. The most important ideal IRB characteristics were timeliness, upholding participants’ rights while also facilitating research, working with investigators to find solutions when there are disagreements, and not allowing biases to affect reviews. For almost all 45 IRB-RAT statements, the rating of the importance of the characteristic was higher than the rating of how much that characteristic was descriptive of IRBs the respondents were familiar with. There was a significant strong correlation between our study’s scores on the ideal IRB characteristics and those of the first and largest published study that employed the IRB-RAT, the US National Validation (USNV) sample in Keith-Spiegel et al. [19].

**Conclusions:**

An understanding of the perceptions held by Singaporean researchers and research support staff on the value that the ethics review process adds, their perceptions of actual IRB functions and characteristics as well as what they view as central to high functioning IRBs is the first step to considering the aspects of the review process that might benefit from improvements. This study provides insight into how our cohort compares to others internationally and highlights strengths and areas for improvement of Singapore IRBs as perceived by a small sample of the local research community. Such insights provide a springboard for additional research and may assist in further enhancing good relations so that both are working towards the same end.

## Introduction

The role of Institutional Review Boards (IRBs), otherwise known as Human Research Ethics Committees (HRECs) or Research Ethics Committees (RECs), in biomedical research remains central to ensuring that research is designed and conducted in accordance with high ethical and scientific standards, that research participants are adequately protected from harms inherent in some research and that their dignity and rights are protected [1]. IRBs were first established by law in the US in the 1970s, partly in response to revelations about harmful research [2, 3]. While the functions of IRBs around the world and across institutions may differ slightly, their general aim is to review proposed research projects involving humans to ensure that the research is ethically justifiable along a number of dimensions before it commences. IRBs will, for instance, assess the potential harms of a proposed research project and ways to minimise such potential harms, the anticipated benefits (to society and/or to participating individuals), the vulnerability of the subject population, the recruitment process, the consent process, researchers’ experienced and expertise, potential conflicts of interest and, in some institutions for some proposals, other aspects of a proposal, such as study design. IRBs also have oversight responsibilities in relation to approved research as well as an educational function in some institutions to which they belong. (See for example: [4–7]).

In recent decades, the research landscape has significantly changed as a result of scientific advances, substantial diversification of research, and increases in the overall volume of biomedical research [8]. As a result, IRBs have made considerable efforts to refine and revise their processes to keep pace and, in many cases, to redefine their role in the research ethics process to extend beyond approval and oversight functions [9]. Nevertheless, research continues to require appropriate ethical oversight provided by IRBs, which, in the context of Singapore, is duly acknowledged in national laws and guidelines [5, 6].

Despite the central role IRBs play in ensuring the ethical conduct of research, it is recognized internationally that IRBs may not be functioning at the high level expected of them [10]; this may be due to the intricate operational facets which characterize them and, in part, due the financial constraints to which some are subject [8, 11], which can impact on the provision of adequate training for IRB members and therefore the quality of review processes and IRB functioning. Moreover, the relationship between IRBs and the research community is often strained and impacted by perceptions and expectations that the research community have of the role and function of IRBs [12–14], which have been shown to diverge from perceptions held by IRBs [15]. For example, researchers view IRB interventions as means of controlling them but IRBs view their involvement as helpful and accommodating; researchers may view IRBs as unfriendly and adversarial as they adopt a stance of interrogation when seeking justifications for aspects of research proposals; researchers may view IRBs as impeding research, as they sometimes forego certain studies for fear of having it rejected but IRBs appear to view this as an inevitable but unintended consequence of processes; researchers view IRBs as exerting considerable power but IRBs do not share this view because they see themselves as simply implementing research regulations; finally, researchers seem to lack an understanding of the research approval machinery which some IRBs view as the reason for delays and lengthy approval processes [15]. Researchers have a range of expectations given that their work is dependent on ethics clearance; they expect, for example, timely reviews, requisite IRB expertise, and IRB ability to appropriately assess ethical issues, including risks and benefits, rather than simply focusing on regulatory compliance [10, 16]. Insight into how researchers view IRB practices can be helpful in overcoming impediments to a good working relationship between the research community and IRBs.

Internationally, a number of empirical studies have examined how biomedical researchers perceive the IRB review process. Systematic literature reviews [10, 17] indicate that these studies vary considerably in terms of sampling characteristics, data collection procedures, survey tools, and analyses, making their results difficult to compare. However, one survey instrument that has enjoyed repeated uptake is the IRB-Researcher Assessment Tool (IRB-RAT), which measures researchers’ perceptions of how 45 IRB characteristics and functions relate to their *ideal* IRB and their *actual* IRB [18]. *Ideal* IRB is to be understood as an IRB that displays features that are most central to enabling researchers to achieve their best work while *actual IRB* is to be understood to relate to IRB features that researchers feel their IRB actually displays. An initial study of 886 US biomedical and social behavioral scientists [19] generated a baseline ‘US National Validation Sample’ (USNV Sample) to validate the IRB-RAT. Subsequent studies have used the IRB-RAT to capture the perceptions of medical researchers at specific institutions [20, 21] and to guide quality improvement of IRB functioning by identifying the areas in which perceived *actual* IRB processes particularly fall short of the identified *ideal* processes [22, 23].

However, to date there has been no systematic attempt to identify and measure how researchers perceive IRB functions and characteristics in Singapore. Indeed, so far only one published study has used the IRB-RAT to measure researchers’ perceptions outside the US [24]. Notably, this is also the only published study originating from the broader Asian region that explicitly explores researchers’ perceptions of IRBs. While there are published studies on IRB processes and characteristics in Asian countries, these tend to describe only the structure and composition of IRBs [25–27] or only measure their operational efficiency [28]. Our study addresses this gap and uses the IRB-RAT to measure how IRB users in Singapore rate their IRB’s review processes and characteristics and what IRB characteristics they would view as improving the quality of IRB functioning. In addition to the IRB-RAT, which collects information about perceptions of the particular IRB researchers are most familiar with, our survey also collected information about the general perception of the impact of IRB review in various areas, such as maintaining public trust in research.

Our study contributes to a general understanding of perceptions around IRB functions and characteristics in Singapore. It aims to generate preliminary evidence that may be useful in guiding institutions and policymakers to improve IRB functioning in Singapore, and to compare Singaporean findings with international findings. The study also provides a platform for further analyses and research to develop a comprehensive understanding of the Singaporean research ethics landscape with the aim of generating more specific suggestions for improvements to local IRB processes and functions in the future. Finally, through the use of the IRB-RAT, the study allows us to compare Singaporean findings with international findings.

## Methods

Data was collected via an anonymous online survey incorporating the IRB-RAT. The survey was launched on the e-Survey platform of the National University of Singapore (NUS) and IP addresses were not collected.

### Survey tool

The survey comprised a welcome message and information about the research followed by four sections, as illustrated in Table 1. The full survey can be found in S1 Appendix.

**Table 1:** Summary of survey structure.

Survey Structure
**Welcome message and information about the research**
**Section 1: Screening questions**
Age: 21+
Involvement in research: Principal Investigator (PI), Co-Investigator (Co-I), research support staff
If inclusion criteria not met–exit survey
**Section 2. General perception of IRB Review**
Rating the impact of the IRB review process on the overall quality of research, establishing/maintaining public trust in research, the protection of research participants, and the scientific validity of research on a 5-point Likert-type scale.
**Section 3. I**nstitutional **R**eview **B**oard **R**esearcher **A**ssessment **T**ool **IRB-RAT**
Rating of 45 items along two dimensions: Ideal & Actual
Example: “An IRB that reviews protocols in a timely fashion”:
***Ideal*:** how important is the item to you to do your best work along a 7-point continuum with 7 = “Absolutely essential” to 1 = “Not important”.
***Actual*:** how well does the item describe the IRB that you are most familiar with in your role as researcher or research support staff, with 7 = “Highly descriptive” to 1 = “Not at all descriptive”, with the additional option of “I don’t know/I have no experience”.
**Section 4. Demographic information**

Section 1 screened potential participants to ensure that they were either a PI/Co-I on a biomedical research project that has undergone IRB review in the past 12 months, or research support staff who had been substantially involved in the drafting of a biomedical research project that had undergone IRB review in the past 12 months. This ensured participation only by individuals who had recent first-hand experience with IRB submissions. Section 2 consisted of questions about the general impact of IRB review in four areas. Section 3 of the survey comprised the core component and was an adaptation of the IRB-RAT, a validated tool serving as a proxy for the quality of IRB functions and characteristics [23]. The survey prompted respondents to indicate on a 7-point Likert-type scale how well 45 statements about IRB characteristics and functions (‘items’) described their ideal and their actual IRB [18]. If participants had experience with more than one IRB, they were asked to respond about only one of the IRBs.

Three variations were made to the original IRB-RAT. First, minor adjustments were made to the wording of some statements to ensure they were applicable to the local context, e.g. ‘federal regulations’ became ‘relevant laws or national guidelines’. The second variation concerned the structure of the published tool. Instead of asking participants to first give views about their *ideal IRB* for all 45 questions and, then, in a second round asking for their views about the *actual* IRB rating across all 45 items, we converted this to a “single-pass” version which contained all 45 items and required consecutive responses, one about the *actual* and one about the *ideal* IRB. The rationale for this change was that the single-pass version would decrease the cognitive load for participants and the time required to complete the survey. The original IRB-RAT, developed by Keith-Spiegel and Koocher allowed for this variation but had not implemented it [29]. The third variation concerned the introduction of the option “I don’t know/ I have no experience”, for responses relating to actual IRBs. This option was included because we judged that researchers and research support staff could not possibly be personally familiar with some internal IRB processes (e.g. “An IRB composed of members who arrive at meetings well-prepared”). We notified the developers of the IRB-RAT that we would be employing their tool and informed them of the modifications that we intended to make.

Section 4 of the survey collected non-identifying demographic data relating to institutional affiliation, IRB membership, type of research engaged in, years of involvement in biomedical research and involvement in international research. The research was approved by the National University of Singapore IRB (S-18-289E) and participants were not reimbursed for their involvement.

### Recruitment

An e-mail invitation to complete the online questionnaire was sent to potential participants via two channels and was open for approximately 6 weeks. The first channel made use of the fact that biomedical research in Singapore may only be conducted under the purview of a Research Institution (RI) that has notified the Ministry of Health of its intention to perform such research. By Singapore law, RIs engaging in biomedical research are required to each appoint a ‘Principle-Person-in-Charge’ (PIC) and a nominated representative, ‘Point-of-Contact (POC)’, and the Ministry of Health maintains a public list of PIC/POCs’ contact details. PICs and POCs at all registered RIs were requested to forward the study invitation to relevant Principal Investigators (PIs), Co-Investigators (Co-Is) and research support staff. During the research period there were 38 registered RIs and the 35 institutions whose PICs/POCs agreed to be contacted to disseminate our research invitation can be found in S1 Table. Two reminder emails were sent. The second recruitment channel involved assistance from Singapore’s College of Clinician Scientists, whose Council forwarded the e-mail invitation to members of the College.

### Piloting

The survey was piloted with three individuals who were either biomedical researchers or research support staff. Participants in the pilot phase provided responses to the survey, time taken for their involvement, general feedback on the ease with which the survey was completed, their interest in completing the survey, and comments on the text provided at the beginning of the survey. Minor adjustments to the layout and the wording in the introduction were made in light of the feedback provided.

### Analysis

Statistical analyses were performed using SPSS v.25, R v.3.5.1 and v.3.6.2. Data were analysed descriptively first, with mean and SD of each item of the IRB-RAT questionnaire reported, excluding the respondents whose answers were *unknown* or *not applicable*. The Wilcoxon Sign Rank test was used to compare the ideal and actual ratings of each IRB-RAT item, while the Mann-Whitney U test was used to compare the ratings of each IRB-RAT item between respondents with different characteristics. The Z-test was used to compare the mean ratings of our cohort with the mean ratings reported in the literature. The correlation between our mean ideal scores and those in the USNV sample [19] and Chenneville and colleagues’ [24] studies was also examined. The correlation between our mean ideal scores and the mean ideal scores in Reeser et al. [20] is not reported because the Reeser et al. sample was not a national sample. However, the correlation between our mean ideal scores and those in Chenneville and colleagues’ study [24] was reported, even though the Chenneville et al. study sample was not a national sample, because it is the only study outside the US to have used the IRB-RAT.

## Results

Among 149 individuals who answered at least 1 screening question, 117 individuals met the inclusion criteria. Ninety-eight individuals started and completed the questions on the general perception of the impact of IRB review and 82 individuals then progressed to start the IRB-RAT. Seventy-one completed it, with the remaining 11 providing responses to only some of the items (Table 2). All responses to the IRB-RAT survey questions were included in the final analysis regardless of whether the respondent completed the rest of the survey. Of the 35 RIs approached, 16 (42%) are represented in our sample with at least one response (S1 Table). The response rate could not be calculated as there was no information on how many researchers and research support staff had received the invitation to participate.

**Table 2:** Responses received per survey section.

Survey section	Respondents who answered at least 1 question in each section	Respondents who completed the section
**Screening question**	149	149
**Section 1.** General perception of impact of IRB review	98	98
**Section 2.** IRB-RAT	82	71
**Section 3.** Demographic information	71	71

Demographic information was only available for 71 respondents who completed the survey since the section was located at the end of the survey. Table 3 lists these demographic details and shows that among the respondents, 53 (75%) were PI/Co-Is while the remaining 18 (25%) were research support staff. The vast majority (93%) were not IRB members. A large proportion of participants (58%) indicated involvement in research involving data only. Respondents could choose multiple affiliations and the most common affiliations clustered around two organisations.

**Table 3:** Characteristics of survey respondents.

Characteristics of Respondents who Completed the Survey (N = 71)		
Member of IRB	Yes	5 (7.0%)
	No	66 (93.0%)
Experience with submission of research protocol to foreign IRBs	Yes	21 (29.6%)
	No	50 (70.4%)
Years in biomedical research	1–3 years	12 (16.9%)
	4–6 years	19 (26.8%)
	7–10 years	23 (32.4%)
	more than 11 years	17 (23.9%)
Type of researcher	PI/Co-I	53 (74.7%)
	Support staff	18 (25.4%)
Type of research^a^	Research involving only data (anonymised or not)	41 (57.7%)
	Research involving only secondary use of previously collected human tissue (anonymised or not)	21 (29.6%)
	Interventional research regulated by the Singapore Health Sciences Authority (HSA), such as clinical trials	21 (29.6%)
	Interventional research that is not regulated by HSA^b^	32 (45.1%)
	Research that is not covered by any of the above categories	18 (25.4%)

^a^ Multiple responses could be selected.

^b^ Health Sciences Authority (Singapore).

Table 4 shows the results from Section 1 of the survey, which asked participants about their general perceptions of the impact of IRB review processes. The 98 participants who completed this section generally thought that the IRB review process has a very positive/positive influence on the following four areas examined: the overall quality of research (59%); establishing/maintaining public trust in research (74%); the protection of research participants (86%); and the scientific validity of research (50%). Few respondents stated that the IRB review process has a negative/very negative impact on these areas. However, a considerable percentage of respondents viewed IRB review processes as having no impact, particularly in relation to contributing to the scientific validity of research (45%) and on the overall quality of research (29%).

**Table 4:** General perception of the IRB review process.

How do you rate the impact of the IRB review process on	N	Very Positive impact (%)	Positive impact (%)	No impact (%)	Negative impact (%)	Very negative impact (%)
a) the overall quality of research	98	3 (3.1%)	55 (56.1%)	28 (28.6%)	8 (8.2%)	4 (4.1%)
b) establishing/maintaining public trust in research		17 (17.3%)	56 (57.1%)	22 (22.4%)	1 (1.0%)	2 (2.0%)
c) the protection of research participants		25 (25.5%)	59 (60.2%)	12 (12.2%)	1 (1.0%)	1 (1.0%)
d) scientific validity of research		4 (4.1%)	45 (45.9%)	44 (44.9%)	3 (3.1%)	2 (2.0%)

The ideal and actual ratings of each IRB-RAT item are summarized in Table 5. The items are ranked by the mean ratings for the ideal IRB in descending order, highlighting which items respondents in our study regarded as most and least important. A wide range of IRB features was viewed as crucial for an IRB, the most important of which included: timely reviews, the promotion of participants’ rights in conjunction with facilitation of research, good collaborative relations with researchers, IRB responsiveness, the absence of reviewer and committee biases, IRB accountability, procedurally and legally knowledgeable members and good record-keeping.

**Table 5:** *Ideal* and *Actual* ratings, ranked in descending order by mean ideal ranking.

Item No.	Item text	Ideal		Actual	
		Mean (SD)	No. of responses on Likert scale	Mean (SD)	No. of responses on Likert scale
3	An IRB that reviews protocols in a timely fashion	6.63 (0.87)	82	3.80 (1.91)	81
41	An IRB that does a good job of upholding participants’ rights while, at the same time, facilitating the conduct of research	6.59 (0.58)	71	4.53 (1.72)	70
8	An IRB that is willing to work with investigators to find mutually satisfying solutions whenever disagreements exist	6.58 (0.73)	79	4.26 (1.86)	74
31	An IRB that responds in a timely manner to investigators’ inquiries about its processes and decisions	6.54 (0.67)	72	4.31 (1.87)	70
4	An IRB whose members do not allow personal biases to affect their evaluation of protocols	6.54 (0.88)	82	5.13 (1.55)	64
26	An IRB that does not use its power to suppress research that is otherwise methodologically sound and in compliance with relevant laws whenever it perceives potential criticism from outside the scientific community	6.50 (0.79)	72	5.07 (1.83)	45
32	An IRB that acknowledges full responsibility for its errors or delays in processing protocols and attempts to correct them as expeditiously as possible	6.50 (0.73)	72	4.11 (2.02)	62
2	An IRB with members who are very knowledgeable about IRB procedures and legal requirements	6.49 (0.82)	82	4.81 (1.54)	77
13	An IRB that maintains complete and accurate records	6.46 (0.82)	76	5.87 (1.14)	75
14	An IRB that is open to innovative approaches to conducting research	6.45 (0.87)	76	4.15 (1.74)	65
22	An IRB that is allocated sufficient resources to carry out functions efficiently and thoroughly	6.44 (0.91)	73	3.88 (2.00)	57
27	An IRB that gives a complete explanation for any required changes to or disapprovals of protocols	6.43 (0.93)	72	4.84 (1.66)	70
11	An IRB that treats investigators with respect	6.41 (0.85)	76	5.31 (1.53)	75
12	An IRB that conducts a conscientious and complete review of protocols	6.41 (0.80)	76	5.53 (1.35)	75
40	An IRB that views its role as being an investigator’s ally rather than as being a hurdle to clear	6.40 (1.07)	72	3.76 (2.18)	68
25	An IRB that offers investigators information to improve the chances of gaining IRB approval	6.40 (0.81)	73	4.60 (1.85)	68
19	An IRB that views protection of human participants as its primary function	6.39 (1.03)	74	5.79 (1.25)	70
28	An IRB that invites investigators to present their position whenever a question or concern about a research protocol arises	6.39 (0.86)	72	4.75 (1.84)	64
23	An IRB that conducts a conscientious, informed analysis of potential benefits weighed against potential risks before making decisions	6.37 (0.84)	73	4.60 (1.63)	67
24	An IRB that holds no preconceived biases against particular research techniques	6.33 (1.14)	73	4.86 (1.67)	56
38	An IRB whose members fully understand and act within the scope of their function	6.31 (0.82)	72	4.91 (1.51)	58
44	An IRB that can competently distinguish exempt from non-exempt research	6.30 (1.15)	71	4.78 (1.77)	65
33	An IRB that is open and pleasant in its interactions with investigators	6.29 (0.97)	72	4.92 (1.84)	72
18	An IRB that takes timely and appropriate action whenever scientific misconduct is alleged	6.27 (1.19)	74	5.49 (1.39)	37
43	An IRB that holds no preconceived biases against particular research topics	6.27 (1.04)	71	4.88 (1.70)	52
5	An IRB that applies appropriately flexible standards regarding voluntary and informed consent requirements (e.g., required wording is less demanding for minimal risk research using competent adult participants)	6.24 (1.05)	82	3.92 (2.01)	76
42	An IRB that is empathetic with the difficulties that can present themselves during the design or conduct of the research	6.21 (1.09)	71	4.03 (1.98)	65
21	An IRB that requires members to abstain from evaluating protocols whenever a real or apparent conflict-of-interest arises	6.18 (1.35)	73	5.72 (1.49)	36
17	An IRB that ensures that at least one member is knowledgeable about the content domain and discipline of submitted protocols	6.18 (1.10)	74	4.70 (1.82)	60
20	An IRB that includes a complete rationale when it denies or mandates changes in a protocol based on criteria that are more stringent than or different from relevant laws or national guidelines	6.18 (1.10)	74	4.34 (1.92)	65
34	An IRB whose Secretariat (or staff member in charge of IRB functions) has a background in conducting research	6.14 (1.01)	72	4.50 (1.87)	52
1	An IRB that is open to reversing its earlier decisions (i.e., willing to carefully listen to investigators’ appeals)	6.13 (1.11)	82	3.96 (1.68)	70
15	An IRB that takes timely action when an investigator has violated the specifications of its rulings	6.11 (1.28)	76	5.57 (1.43)	47
36	An IRB that requires its Chair be an experienced investigator	5.99 (1.20)	72	5.18 (1.41)	49
35	An IRB that monitors the progress of each approved research project in line with relevant laws and national guidelines	5.92 (1.25)	72	5.12 (1.52)	66
30	An IRB that offers investigators opportunities to be educated about relevant laws and national guidelines	5.86 (1.14)	72	4.21 (1.91)	63
10	An IRB that provides a comprehensive training program for its new members	5.85 (1.28)	79	4.47 (1.72)	55
6	An IRB that recognizes when it lacks sufficient expertise to evaluate a protocol and seeks an outside evaluator	5.81 (1.44)	79	4.23 (1.99)	52
7	An IRB that shows considerable evidence that the advancement of science is part of its mission	5.80 1.36)	79	3.85 (1.86)	68
29	An IRB that offers consultation during the development of research protocols or grant applications	5.74 (1.41)	72	4.0 (2.12)	54
37	An IRB that has a diverse membership (i.e., includes women, minorities and both junior and senior members of the institution)	5.74 (1.53)	72	4.74 (1.71)	43
45	An IRB composed of members who arrive at meetings well-prepared	5.70 (1.56)	71	5.57 (1.25)	30
16	An IRB that is composed primarily of highly competent investigators	5.61 (1.27)	74	4.55 (1.76)	56
9	An IRB that offers editorial suggestions regarding consent documents and protocols (e.g., typos, grammar, clarity)	4.91 (1.73)	79	4.65 (1.83)	75
39	An IRB that is composed of more than one lay person	4.51 (1.96)	72	4.83 (1.58)	40

*Note*. The number of responses for the “I don’t know/ I have no experience” option for each IRB-RAT item is equal to the number of responses on the Likert scale for the ideal dimension of that item subtracted by the number of responses on the Likert scale for the actual dimension of that item.

There were fewer responses in the *actual* dimension than in the *ideal* dimension for all items except item 33 (“An IRB that is open and pleasant in its interactions with investigators”), as participants could select the option “I don’t know/ I have no experience” for *actual* (but not for *ideal*) items. For some items, the number of participants who selected this option was significant. For instance, 41/71 respondents who answered the question did not know whether the members of the IRB that they were most familiar with arrived at meetings well prepared (item 45) and 37/73 respondents who answered the question did not know whether the IRB members would abstain from evaluating protocols whenever a real or apparent conflict of interest arose (item 21).

**Fig 1:**
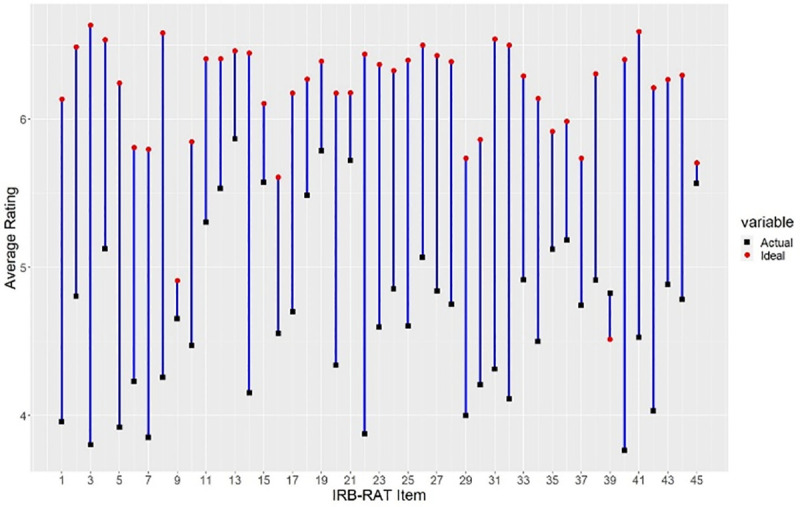
Mean ideal and actual ratings for each IRB-RAT item.

Fig 1 below plots the mean ideal and mean actual ratings. The importance of an ideal IRB possessing an item was rated higher than the actual IRB performance on all items except for item 39 (“An IRB that is composed of more than one lay person”). Item 39 also had the lowest mean ideal rating. This suggests that respondents may not see much value in having lay people on IRBs.

Ideal and actual scores for each of the 45 IRB-RAT items were compared, and the results show that all items in our study had significantly higher mean ideal scores than the mean actual scores, except items 9 and 39 for which the difference was not statistically significant (S2 Table contains further details).

**Fig 2:**
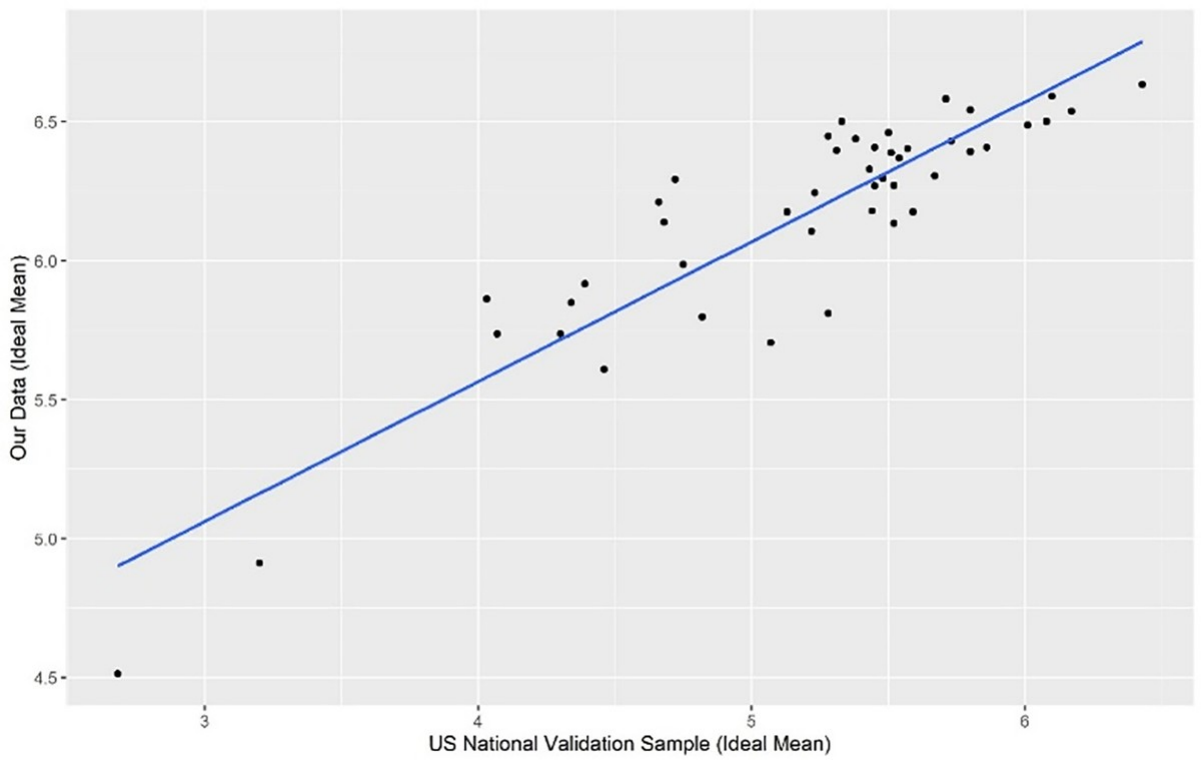
Plot of the ideal mean found in our study against that in the USNV Sample in Keith-Spiegel et al. [19].

We compared our ideal scores with those in Keith-Spiegel and colleagues’ USNV sample [19], Reeser et al. [20] and Chenneville et al. [24] and compared our actual scores and the difference between the ideal and actual scores with those in Reeser et al. [20] and Chenneville et al. [24]. We found a significant strong correlation between the mean ideal scores in our study and those in the USNV sample (p < 0.001, Spearman’s Rho = 0.817, df = 43). A plot of our ideal responses against those of Keith-Spiegel et al. [19] is shown in Fig 2 below. This result is consistent with findings in Hall et al. [23].

Moreover, three of the top five ranked ideal items (3, 4, 41) and three of the last five ranked ideal items (9, 37, 39) reported in the USNV sample were the same as those in our data. For example, timely reviews, IRB commitment to both participants’ rights and research, and not allowing personal biases to affect the evaluation of protocols featured as the most important ideal IRB features for both our respondents and the respondents in the USNV sample (S4 Table). Similarly, both the Singaporean cohort in our study and the USNV sample [19] viewed lay membership and diversity of membership as well as editorial suggestions from the IRB as some of the least important features of an IRB (S5 Table). Our ideal scores for all 45 IRB-RAT items were significantly higher than those in the USNV sample [19] (S6 Table). The range of the difference between the mean ideal scores in our study and the mean ideal scores in the USNV sample [19] was 1.63.

Forty-one *actual* IRB-RAT items in our study scored significantly lower than those in Reeser and colleagues’ study [20]. However, only 17 ideal items showed significant difference between our data and those in Reeser and colleagues’ study [16].

With regard to Chenneville and colleagues’ study [24], only six ideal items and ten actual items differed significantly. The correlation between the mean ideal scores in our study and the mean ideal scores in Chenneville and colleagues’ study [24] was weak (p < 0.001, Spearman’s Rho = 0.476, df = 43). Only one of the five most important ideal items (item 4) and two of the five least important ideal items (items 9 and 39) were the same in both our study and Chenneville and colleagues’ study [19, 24].

Respondents in Chenneville and colleagues’ study [24] reported a significantly smaller expectation gap (i.e. the difference between the ideal and actual IRB ratings) than respondents in our study in a number of areas. A smaller expectation gap indicates that an IRB more closely met the respondents’ expectation. In Chenneville and colleagues’ study a significantly smaller expectation gap was observed in areas that would promote science and research, for example: *being open to innovative ways of performing research* (item 14); *being viewed as an ally rather than as a hurdle to clear* (item 40); *showing considerable evidence that the advancement of science is part of its mission* (item 7); *being willing to work with investigators to find mutually satisfying solutions whenever disagreements exist* (item 8); *ensuring that at least one member is knowledgeable about the content domain and discipline of submitted protocols* (item 17). In these areas, respondents in our survey perceived IRBs as falling shorter of their expectations compared with respondents in Chenneville and colleagues’ study [24]. On the other hand, respondents in our study had a significantly smaller expectation gap, indicating that IRBs more closely met their expectations in adhering to certain rules and formalities compared with what was reported in Chenneville and colleagues’ study [24]. The S7 Table summarizes the different areas in which we observed expectation gaps in these two studies. Compared to that of Reeser and colleagues’ study [20], the mean difference between the ideal and actual IRB ratings in our study was significantly greater for 38 items. Further details can be found in S3 Table.

Table 6 groups the IRB-RAT items along two rating dimensions: their *relative importance* as measured by their ideal scores and their *relative performance* as measured by the paired difference between the ideal and actual scores. IRB performance in this part of the analysis was operationalized using the paired difference rather than the actual scores because the former better reflects which items require improvement. Our analysis used paired difference to reduce the effect of confounding variables. For example, respondents could not know how an actual IRB rates on an item for which they had no direct knowledge because they were not present during IRB deliberations. This may have affected how important they thought that factor was in an ideal IRB.

**Table 6:** The classification of IRB-RAT items according to performance and importance.

	At or Below Average Importance (‘low importance’)	Above Average Importance (‘high importance’)
**Paired difference (Ideal-actual) at or below average [performance at or above average]**	**Category 1**	**Category 2**
	(9)* An IRB that offers editorial suggestions regarding consent documents and protocols (e.g., typos, grammar, clarity)	(4) An IRB whose members do not allow personal biases to affect their evaluation of protocols
		(11) An IRB that treats investigators with respect
	(10) An IRB that provides a comprehensive training program for its new members	
		(12) An IRB that conducts a conscientious and complete review of protocols
		(13) An IRB that maintains complete and accurate records
		(18) An IRB that takes timely and appropriate action whenever scientific misconduct is alleged
	(15) An IRB that takes timely action when an investigator has violated the specifications of its rulings	
		(19) An IRB that views protection of human participants as its primary function
	(16) An IRB that is composed primarily of highly competent investigators	(21) An IRB that requires members to abstain from evaluating protocols whenever a real or apparent conflict-of-interest arises
	(35) An IRB that monitors the progress of each approved research project in line with relevant laws and national guidelines	(24) An IRB that holds no preconceived biases against particular research techniques
		(26) An IRB that does not use its power to suppress research that is otherwise methodologically sound and in compliance with relevant laws whenever it perceives potential criticism from outside the scientific community
	(36) An IRB that requires its Chair be an experienced investigator	
	(37) An IRB that has a diverse membership (i.e., includes women, minorities and both junior and senior members of the institution)	(28) An IRB that invites investigators to present their position whenever a question or concern about a research protocol arises
		(33) An IRB that is open and pleasant in its interactions with investigators
	(39) An IRB that is composed of more than one lay person	(38) An IRB whose members fully understand and act within the scope of their function
	(45) An IRB composed of members who arrive at meetings well-prepared	(43) An IRB that holds no preconceived biases against particular research topics
		(44) An IRB that can competently distinguish exempt from non-exempt research
**Paired difference (Ideal-actual) above average [performance below average]**	**Category 3**	**Category 4**
	(1) An IRB that is open to reversing its earlier decisions (i.e., willing to carefully listen to investigators’ appeals)	(2) An IRB with members who are very knowledgeable about IRB procedures and legal requirements
		(3) An IRB that reviews protocols in a timely fashion
		(5) An IRB that applies appropriately flexible standards
		regarding voluntary and informed consent requirements (e.g., required wording is less demanding for minimal risk research using competent adult participants)
	(6) An IRB that recognizes when it lacks sufficient expertise to evaluate a protocol and seeks an outside evaluator	
		(8) An IRB that is willing to work with investigators to find mutually satisfying solutions whenever disagreements exist
		(14) An IRB that is open to innovative approaches to conducting research
	(7) An IRB that shows considerable evidence that the advancement of science is part of its mission	
		(17) An IRB that ensures that at least one member is knowledgeable about the content domain and discipline of submitted protocols
		(20) An IRB that includes a complete rationale when it denies or mandates changes in a protocol based on criteria that are more stringent than or different from relevant laws or national guidelines
	(29) An IRB that offers consultation during the development of research protocols or grant applications	
		(22) An IRB that is allocated sufficient resources to carry out functions efficiently and thoroughly
	(30) An IRB that offers investigators opportunities to be educated about relevant laws and national guidelines	(23) An IRB that conducts a conscientious, informed analysis of potential benefits weighed against potential risks before making decisions
		(25) An IRB that offers investigators information to improve the chances of gaining IRB approval
	(34) An IRB whose Secretariat (or staff member in charge of IRB functions) has a background in conducting research	(27) An IRB that gives a complete explanation for any required changes to or disapprovals of protocols
		(31) An IRB that responds in a timely manner to investigators’ inquiries about its processes and decisions
		(32) An IRB that acknowledges full responsibility for its errors or delays in processing protocols and attempts to correct them as expeditiously as possible
		(40) An IRB that views its role as being an investigator’s ally rather than as being a hurdle to clear
		(41) An IRB that does a good job of upholding participants’ rights while, at the same time, facilitating the conduct of research
		(42) An IRB that is empathetic with the difficulties that can present themselves during the design or conduct of the research

* N.B. The item number of each IRB-RAT item is in parentheses. Within each category, items are ordered by item number.

The left column in Table 6 shows items of ‘low importance’, understood as items whose ideal score is at or below overall (weighted) average of the ideal scores and the right column shows the ‘high importance’ items. The top half of this quadrant shows items whose performance was at or above average compared to the overall (weighted) average of the paired difference between the ideal and actual scores, while the bottom half of the quadrant shows items whose performance was below the (weighted) average. This method of analysis is derived from Hall and colleagues’ 2015 study [23].

Category 4 in Table 6 consists of items that were regarded as *above average importance* but *below average performance*. These 16 items include the 5 lowest performing IRB-RAT items, as measured by the paired difference between ideal and actual scores (items 3, 40, 22, 32, and 5). This suggests that attention should be paid to the items in category 4 as items most in need of improvement. Further analysis of these 16 items in category 4 is taken up in a separate forthcoming publication.

There was no statistically significant effect of experience of submissions to foreign IRBs, years of experience in biomedical research, or type of researcher (PI/Co-I vs. research support staff). Furthermore, the effect of IRB membership was indeterminable because only five participants were IRB members. However, there was a statistically significant difference between respondents who had experience performing data-only research and respondents without experience in data-only research for 14 items (see S8 Table.) Most of these items (12 of 14) concerned ratings on the actual IRB. In all of the 12 items on the actual IRB, respondents with experience in data-only research rated the actual IRBs lower than respondents who did not have experience performing data-only research. On the other hand, respondents with experience in data-only research provided significantly higher ratings for the two items that concerned ideal IRBs: a*n IRB that views its role as being an investigator’s ally rather than as being a hurdle to clear* (item 40); and an *IRB that is empathetic with the difficulties that can present themselves during the design or conduct of the research* (item 42).

S1 and S2 Figs show the distribution of ideal ratings on IRB-RAT items 40 and 42 respectively, grouped by respondents with and without experience performing data-only research. In S1 Fig we see that respondents with experience in data-only research selected only ratings 6 (27%) and 7 (73%). In contrast, respondents without experience in data-only research selected a wider range of ratings, and the proportion of those selecting ratings 6 (23%) and 7 (53%) was lower. In S2 Fig we show that of all respondents with experience in data-only research, 34% selected rating 6 and 59% selected rating 7. In contrast, of all respondents without experience in data-only research, 30% selected rating 6 and 40% selected rating 7.

## Discussion

This is the first study conducted in Singapore that aimed to gain an understanding of a. how local researchers and research support staff view the impact of IRB review generally; b. how they perceive the IRBs they are in contact with; and c. which IRB features and functions they view as important for IRBs to display, as these impact on their work. Despite the small sample and the inability to ascertain the total number of such researchers and research support staff in Singapore, the findings are important as they provide the only insight we have into perceptions of the characteristics and functioning of Singaporean IRBs. Due to the nature of the findings, IRBs could still consider improvements in the areas identified, particularly because the findings are supported by international literature. This is an opportunity for Singaporean IRBs to pause and consider how they could more generally attend to perceptions about their role and function in the research ethics process and the value of cohesive working relationships.

There were at least four key insights that can be gleaned from our study. Firstly, there was an overall positive perception of the impact of the ethics review process on a number of dimensions. Secondly, gaps between ideal and actual scores in our results highlight to IRBs and institutions where they may want to focus reform efforts. Thirdly, where there are strong areas of overlap between perspectives among Singaporean researchers and those in the US, the solutions to concerns adopted in the US may be particularly worthy of attention for possible adaptation in Singapore. And finally, appropriate reforms to IRB processes to address researchers’ concerns may be substantially different for data-only research compared with other forms of research, reflecting the divergent priorities and needs of different types of researchers. We will now discuss each of these insights in more detail.

### Perceptions of ethics review as value-adding

In terms of perceptions of the general impact of IRB reviews, the majority of respondents reported that IRB review has an overall positive impact. This generally positive attitude towards IRB review provides confidence that there is a general appreciation in this small cohort of researchers and research support staff of the intended role and purpose of IRBs.

However, a sizeable proportion of respondents felt that IRB review has no impact and an even smaller proportion felt that IRB review has a negative impact on the quality of research; establishing and maintaining public trust in research; the protection of research participants; and on the scientific validity of research. The perspectives of this minority who viewed IRB review negatively, while not representative of the entire Singaporean research community, are nevertheless cause for serious reflection. There is now considerable research ethics guidance available and awareness of legislation which regulates the conduct of research via IRBs and other relevant bodies but the justification for such controls may not be apparent to all. For example, unless researchers are familiar with the historical and scientific context within which research ethics developed, it is possible that the extensive focus on human participant protections, to give one example, may appear excessive and even obstructive. On the other hand, some of the lowest performing *actual* IRB-RAT items, which were viewed as important in this study, related to IRBs’ level of content knowledge and ability to conduct informed analyses. Therefore, negative views of the limited value that the ethics review process adds may also reflect these participants’ views of committee members’ competence; that is, their ability to correctly identify ethical issues that require consideration and suggest amendments that appropriately address these issues rather than focusing on unnecessary changes to protocols. Such issues echo researcher concerns in other parts of the world [16]. If some in the research community fail to see the value in the review process, they are more likely to view it as a hurdle to clear rather than appreciating both the underlying reasons requiring review and the potential benefits arising from quality reviews. Such neutral and negative perceptions of the general value of ethics review have the potential to influence other researchers and adversely impact on researcher-IRB relationships [11, 13].

### Highlighting potential areas for improvement

Concentrating more specifically on the findings of the IRB-RAT survey itself, IRBs in Singapore may wish to focus on the expectation gaps identified in this study. Identifying wider expectation gaps may help highlight areas where there is greater perceived need for reform while smaller expectation gaps may reveal areas where systems are perceived to be functioning in accordance with researchers’ needs and expectations. For the purpose of generating evidence to guide improvements to IRB review processes and functioning in Singapore, the results listed in Table 6 hold important insights, especially the areas where local IRBs are viewed as underperforming but which the respondents regarded as important features and functions of an IRB. The five areas identified by our participants as requiring most attention cover a range of procedural and substantive IRB characteristics and functions and align with concerns expressed in the literature.

#### Timeliness

Timely review of protocols was the issue identified as requiring most attention, an issue repeatedly identified in a range of research areas internationally [10, 19, 22, 30] including disaster research where timeliness is of central importance [31]. Timely reviews are not simply a matter of inconvenience for researchers; they create significant delays in accessing findings that are of potential great public benefit [32]. In addition, the detrimental impact IRB delays may have on interpersonal relationships has multiple dimensions, as members of IRBs are also members of the research community. It should, however, also be acknowledged that researchers also contribute to the protracted review period with delays in responding to IRB requests for clarification or amendment of the protocol [28].

#### IRBs as allies

Of great importance to the conduct of research but lacking in these participants’ experience with IRBs was a collaborative and supportive relationship with IRBs, an issue also identified elsewhere [11, 15]. When researchers and IRBs do not view themselves as sharing a common goal in promoting quality research, this not only frustrates important interpersonal relationships but may ultimately impact negatively on scientific advances and clinical innovation [33]. IRBs have the potential to play a significant role in guiding researchers, in a collaborative rather than authoritarian way, to achieve ethically, legally, and scientifically sound research thus contributing to greater societal benefits but also to more cohesive working relationships.

#### Need for IRB resources

In order to be in a position to guide the research community and function efficiently, IRBs require sufficient resources, an issue acknowledged by our participants and others [10]. Unless resources are available to ensure that adequate sustained training is available, it is unlikely that quality ethics reviews can be achieved. An environment where superficial reviews that focus on bureaucratic requirements yet overlook important issues of ethical import is likely to develop in the absence of appropriate training for IRB members.

#### Truthfulness and accountability

It is unlikely that IRBs could ever avoid making errors but our participants felt that truthfulness and accountability for IRB errors (and processing delays) were important but lacking. Such perceived deficiencies also impact on interpersonal relationships and could be perceived as a lack of respect towards researchers. Making improvements in this area would attend to issues of justice but would perhaps require an attitudinal shift regarding the roles and functions of IRBs within institutions. For example, IRBs would need to view one of their primary roles as supporting researchers, in line with the issue raised above.

#### IRB competence

The fifth area that our participants viewed as most important yet most deficient related to IRB competency levels; more specifically, to IRBs being knowledgeable and discerning regarding the appropriate yet flexible application of informed consent requirements. Arguably, one of the greatest challenges for researchers and IRBs alike is striking the right balance between adhering to consent requirements that demonstrate appropriate consideration of and respect for research participants without being excessively burdensome and ultimately ineffective. IRBs have received harsh criticism regarding their perceived shifting objectives: “Rather than protecting research subjects from harm, they now seem especially focused on protecting universities and research centers.”p. 611 [34].

The discrepancy between these participants’ perceptions of IRB functions and characteristics of great value where IRBs are deficient may be important for IRBs to consider, particularly if they value a collaborative relationship of trust with the research community. As previously indicated, these findings have been subjected to additional analysis and will be presented in a separate forthcoming paper.

### International comparison

Unlike several other published studies that have employed the IRB-RAT, our recruitment pool was not limited to a single institution or health system nor to researchers only; we aimed to include any biomedical researcher or research support staff within Singapore. The only other published study that reports national data is the USNV sample [19], which involved biomedical and social behavioural scientists from a wide range of institutions. The high degree of correlation between the mean ideal scores in our study and those in the USNV study [19], as opposed to the lower degree of correlation between the mean ideal scores in our study and those in the Chenneville et al. study [24], are important to consider given that much guidance on IRB operations and functioning comes out of the US and may be relevant to IRB functioning in Singapore. If researcher perceptions are similar, it may be that solutions to address the gaps are also similar. We will therefore now discuss and highlight the implications of several areas of overlap between our study and the USNV sample.

In the assessment of functions and characteristics that are most important for the ideal IRB, the views of our respondents were broadly in line with the USNV sample [18, 19], indicating some apparent degree of alignment between Singaporean and US researcher perspectives on what constitutes an ideal IRB. Timeliness of IRB review processes was ranked as the most important IRB characteristic in both studies, reflecting researchers’ desire to avoid unnecessary delays in carrying out their research. Indeed, in Singapore, two of the top five ideal features of an IRB related to timeliness. We have already raised some implications arising from delays in the ethics review process above. In addition to the practicalities of receiving timely responses and ethics review outcomes, procedural timeliness carries an important ethical dimension; research is publicly funded and extensive delays in the approval process arising from deficiencies in IRB processes and functions contribute to wasting valuable and limited public funds [32]. Another similarity between the two studies included the fact that both US and Singaporean cohorts rated balancing protecting participant rights whilst also facilitating research very highly suggesting that researchers are not eager to ignore research participants’ interests, but prefer a proportionate approach to ethics review.

Conversely, in both studies, items relating to having IRBs that are diverse and contain multiple lay members were regarded as less important. In fact, for our study, the only item where the actual average rating exceeded the ideal average rating was item 39, which states “an IRB that is composed of more than one lay person.” This may indicate that researchers in both Singapore and the US regard lay persons as being unqualified to evaluate their research proposals. It might also show lack of understanding about why the inclusion of lay members is often mandated, i.e. to voice concerns of potential research participants, who are themselves likely to be non-scientists, and to assist in ensuring that information that is conveyed to potential participants is accessible to laypersons [35]. The absence of scientific training is, in this regard, the asset of a lay person. Another commonly low-ranked ideal item was the IRB’s role in offering editorial suggestions to protocols and participant information sheets. Given that IRBs are tasked with evaluating ethical and regulatory aspects of a study, correcting typographical and grammatical errors may be seen as trivial or irrelevant to their remit even though errors do, in fact, impact on the accuracy of the message conveyed, readability, and comprehension of a text. Respondents in both studies did, however, value IRBs’ input to overcome disagreements, (3^rd^ highest ideal score in our study, 10^th^ highest ideal scores in the USNV sample) which may indicate that they viewed such collaboration as essential to timely approvals.

There were also a few relevant differences between our study and other studies. Our ideal scores for all 45 IRB-RAT items were higher than those of Keith-Spiegel and colleagues’ study [19]. While both cohorts valued similar items most highly, the Singaporean cohort seemingly attached greater importance to all listed IRB features. At the same time, the majority of our study’s ratings on the actual performance of an IRB were significantly lower than those of Reeser and colleagues’ study [20]. On the surface, this would indicate that our cohort viewed the quality of local IRBs as lower. However, we do need to bear in mind the different composition of respondents in the various surveys, and the difference in contextual factors. For instance, 64% of all respondents in Keith-Spiegel and colleagues’ study [19] were social/behavioural scientists, while all of our respondents were involved in biomedical research. Furthermore, the respondents in Reeser and colleagues’ study [20] came from a single, not-for-profit medical centre in rural United States, while our respondents were spread out across multiple institutions in an Asian city-state. The fact that our study was conducted more than 10 years after other studies [19, 20] may also have contributed to the difference in results.

### Type of research and respondents

An unexpected finding was that there was no statistically significant difference in responses from those leading the research (PIs/Co-Is) and those assisting with research protocols and submissions (research support staff). This is in contrast to other studies that have observed a difference [20]. Our findings suggest that in Singapore both researchers and research support staff have similar perceptions when it comes to the actual performance of IRBs and also consider the same kinds of characteristics and functions identified as ideal for an IRB as being important. This is a positive finding, as such concordance is beneficial to the development of research proposals for submission to IRBs.

The relationship between the type of research conducted and the assessment of the actual performance of the IRB also merits consideration, particularly given the increasingly prominent role big data is playing in research and development around the world. We hypothesized that researchers who conduct research involving data only would have a more negative view of the actual functioning of their IRB since they may not as readily see the value in IRB review given the use of data rather than interacting directly with participants. There is evidence, for example, that some researchers do not consider local ethics approval a requirement when accessing previously collected data, such as clinical trial data deposited in the ClinicalStudyDataRequest.com repository [36]. Our findings may support this hypothesis. Respondents with experience in data-only research rated actual IRB performance significantly lower than respondents without experience in data-only research in areas such as: IRB members being knowledgeable about IRB procedures and legal requirements; IRBs being allocated sufficient resources to carry out functions efficiently and thoroughly; and IRBs showing considerable evidence that the advancement of science is part of their mission. On the other hand, respondents with experience in performing data-only research placed significantly higher importance on an IRB which views itself as an ally rather than as a hurdle for researchers and an IRB which is empathetic with difficulties that occur during the design or conduct of research.

The results suggest that researchers who have experience performing data-only research may have different perceptions on some aspects of IRB functioning compared to researchers who do not have experience performing data-only research. These findings may relate to perceived challenges IRBs face with the methodological and technical complexities involved in some data-only research, the complexity in identifying the ethical and legal issues in such research, and in applying guidelines that do not fully consider new research methods. These perceptions may also point to the perceived need for IRB members to receive appropriate training in related areas such as data science. A scoping review conducted in 2018 identified such issues as being considerable challenges for IRBs in relation to the review of big data in health research [37]. The importance that the respondents in our study placed on a close collaborative relationship with IRBs may point to what they view as the way forward to communicating the intricacies of their research and finding mutually acceptable solutions. Additional research in this area may be useful in further clarifying the identified difference between those conducting data-only research and those engaging with research participants directly.

Our research also led to the improvement of the IRB-RAT, which is relevant for those wishing to employ the tool in future studies. The original design did not give participants the option to select “I don’t know” in response to questions about how their IRB was faring along the actual dimension. Although the IRB-RAT developers raised the possibility that such an option could be added [18], it was not included in previously published studies. Our results confirm that for a number of the IRB-RAT items a substantial proportion of respondents could not provide a response on the 7-point Likert-type scale, as several of these items presuppose either a degree of familiarity with the inner workings of specific IRBs or intimate knowledge of the features of specific IRBs that respondents could not possibly have first-hand unless they were members of that IRB.

## Limitations

Limitations of our study should also be acknowledged. The response rate could not be calculated, which means that we cannot calculate the extent to which our results are representative. A related point is that even though recruitment was aimed at researchers and research support staff from all biomedical RIs in Singapore, participants came from only 16 of these. Finally, an issue which impacted on recruitment numbers was the fact that we did not have direct access to researchers’ contact details to recruit directly but rather relied on third parties to disseminate the invitation to participate. This method always yields lower participation rates.

## Conclusions

Respondents in this study generally consider ethics review as a process that adds value in key areas such as the quality of research, establishing and maintaining public trust in research, the protection of research participants, and the scientific validity of research. There remains, however, some work to be done to improve such perceptions, as a proportion do not consider the ethics review process as value-adding; in fact, a small minority view the process as detrimental to research.

Singaporean respondents reported highly valuing IRB functions and characteristics that positively impact on the efficiency of review, including timeliness and a balance between protecting research participants and facilitating the conduct of research, as well as IRB willingness to work with investigators to find mutually acceptable solutions where disagreements emerge. Conversely, matters relating to IRB composition were rated as being of relatively less importance. We established that these Singaporean researchers and research support staff hold similar views to each other as well as similar views to those surveyed in some international studies about the most important features and functions of an ideal IRB; they also hold similar perceptions about the IRBs they most deal with. There was a greater gap between our respondents’ conception of an ideal IRB and the reality of the IRBs they deal with. Local differences also emerged between those dealing with data-only research and those engaged with recruitment of participants, which is an area that warrants additional research given the increasing dependence on big data, particularly in Singapore.

This research is a first step in shedding light on researcher and research support staff perceptions. While there are current gaps, the ideal would be for researchers to view IRBs as a resource rather than an obstacle and for IRBs to view researchers as valuable contributors to scientific, medical, and social progress. Further, it would be mutually beneficial for IRBs to view researchers as a group whom they should assist to improve research protocols both to better protect research participants but also to enhance the scientific integrity of research. An added benefit of such collaborative efforts would also be a reduction in approval timelines, an issue of prime importance to this and other research communities internationally.

## Supporting information

S1 Appendix Study tool. (DOCX)

S1 Table Research Institutions that have notified the Ministry of Health, Singapore, of their operations (as at 4 September 2018). (DOCX)

S2 Table Paired difference between our ideal and actual scores. (DOCX)

S3 Table Comparison between our paired difference of ideal and actual scores with the difference of ideal and actual scores in other studies. (DOCX)

S4 Table The 5 most important ideal IRB characteristics. (DOCX)

S5 Table The 5 least important ideal IRB characteristics. (DOCX)

S6 Table Ideal and actual scores from other studies and whether they differ significantly from our study cohort. (DOCX)

S7 Table Comparison in expectation gap between Chenneville et al. [24] study and our study. (DOCX)

S8 Table Items where respondents with experience in data-only research score significantly differently from those without experience in data-only research. (DOCX)

S1 Fig (DOCX)

S2 Fig (DOCX)

## Abbreviations

IRB: Institutional Review Board (i.e. Human Research Ethics Committee)
PIC: Principle-Person-in-Charge
POC: Person-of-Contact
RI: Research Institution
USNV sample: US National Validation sample
