# Correction: Perspectives of Singaporean biomedical researchers and research support staff on actual and ideal IRB review functions and characteristics: A quantitative analysis

**DOI:** 10.1371/journal.pone.0248613

**Published:** 2021-03-10

**Authors:** Markus K. Labude, Liang Shen, Yujia Zhu, G. Owen Schaefer, Catherine Ong, Vicki Xafis

The email addresses for the corresponding authors are incorrect. Markus K. Labude’s email address is: markus.labude@nus.edu.sg. Vicki Xafis’s email address is: vicki.xafis@nus.edu.sg.

There are errors in the Author Contributions. The correct contributions are:

Conceptualization: Vicki Xafis, Markus K. Labude, G. Owen Schaefer, Liang Shen, Catherine Ong.

Data curation: Yujia Zhu.

Formal analysis: Liang Shen, Yujia Zhu.

Investigation: Markus K. Labude, Vicki Xafis, G. Owen Schaefer.

Methodology: Vicki Xafis, Markus K. Labude.

Project administration: Markus K. Labude, Vicki Xafis.

Supervision: Vicki Xafis.

Visualization: Markus K. Labude, Yujia Zhu, Vicki Xafis, G. Owen Schaefer, Liang Shen, Catherine Ong.

Writing–original draft: Markus K. Labude, Yujia Zhu, Vicki Xafis, G. Owen Schaefer, Liang Shen.

Writing–review & editing: Vicki Xafis, Markus K. Labude, Yujia Zhu, G. Owen Schaefer, Liang Shen, Catherine Ong.
