# Supplementary material for: Perspectives of Singaporean biomedical researchers and research support staff on actual and ideal IRB review functions and characteristics: A quantitative analysis
Source: PLoS One. 2020 Dec 31;15(12):e0241783. doi: 10.1371/journal.pone.0241783 (PMC7774925; doi:10.1371/journal.pone.0241783)
Supplement: S1 Appendix — (DOCX) [file pone.0241783.s001.docx]

**S1 Appendix.**

**Study Tool**

1. **Welcome message and information about the research**

This is a collaborative research project led by the NMRC-funded initiative *Science, Health and Policy-relevant Ethics in Singapore* (SHAPES), at the Centre for Biomedical Ethics, NUS. The study team comprises Dr Vicki Xafis, Mr Markus Labude, Dr Owen Schaefer, Dr Shen Liang, and Asst/Prof Catherine Ong.

This research aims to collect responses from researchers and research support staff regarding actual and ideal IRB processes and functions and to measure differences between the two. The questionnaire you will complete is a modified version of a validated tool known as the IRB-RAT. The tool has been used internationally so we will be able to compare the Singapore outcomes with international studies.

The responses will be anonymous and the IP address will not be collected to guarantee complete anonymity. In addition, we do not seek to collect any demographic data that will identify you. As a result, this research is of minimal risk.

There is no direct benefit to you by participating. However, the research is hoped to benefit the Singaporean research community and assist IRBs in gaining an understanding of how current processes are perceived. The study results may ultimately lead to improvements in IRB review processes and functions.

By responding to the survey, you agree to be involved in the research. Withdrawal is not possible, as the survey is anonymous. We encourage you to respond to all questions so there are no missing data.

If you have any queries about the research, please contact Dr Vicki Xafis at [vicki.xafis@nus.edu.sg](mailto:vicki.xafis@nus.edu.sg).

There are 54 questions in all and the survey takes approximately 20 minutes to complete.

**The research team thanks you very much for your participation!**

In the event that you are not able to complete the survey in one sitting please click the SAVE button. You will be provided with a unique URL that you can record and return to when you are able. However, please note that the survey will close one month from when you received the invitation to participate, and after that point it will not be possible to return to the survey.

1. **Screening Questions**
2. Are you at least 21 years of age?

Yes-No

*(If response is ‘No’ to either question🡪 exclusion criterion, end of survey)*

1. Please tick one option that describes your involvement in research:
2. I am a PI/Co-I on at least one biomedical research project that has undergone IRB review in the past 12 months.
3. I work as research support staff (i.e. research coordinator, research associate, etc.) and have been substantially involved in the drafting of at least one biomedical research project that has undergone IRB review in the past 12 months.

(C) Neither (A) nor (B).

*(If response is ‘C’ 🡪 exclusion criterion, end of survey)*

1. **General perception of IRB review**

**Question:** How do you rate the impact of the IRB review process on…

1. the overall quality of research?

| Very positive impact | Positive impact | No impact | Negative impact | Very negative impact |
| --- | --- | --- | --- | --- |

1. establishing/maintaining public trust in research?

| Very positive impact | Positive impact | No impact | Negative impact | Very negative impact |
| --- | --- | --- | --- | --- |

1. the protection of research participants?

| Very positive impact | Positive impact | No impact | Negative impact | Very negative impact |
| --- | --- | --- | --- | --- |

1. scientific validity of research?

| Very positive impact | Positive impact | No impact | Negative impact | Very negative impact |
| --- | --- | --- | --- | --- |

1. **Researcher Assessment Tool**

**Instructions:** Several characteristics and activities of IRBs are reflected in the 45 items below. How important is each one to you in *your* work?

- **First** rate how important each item would be to you to do your *best* work along a 7-point continuum with 7= “Absolutely essential” to 1= “Not important”.
- **Then**, rate how well that item describes the IRB that you are most familiar with in your role as researcher or research support staff, with 7= “Highly descriptive” to 1=“Not at all descriptive”. If you lack sufficient knowledge or experience to answer any of these descriptive items, please select “I don’t know/ I have no experience”.
- If you have experience with more than one IRB, please respond about only one of the IRBs.

In the event that you are not able to complete the survey in one sitting please click the SAVE button. You will be provided with a unique URL that you can record and return to when you are able.

1. **An IRB that is open to reversing its earlier decisions (i.e. willing to carefully listen to investigators’ appeals)**

7 6 5 4 3 2 1

Essential Moderately Important Not important

I don’t know/
I have no experience

7 6 5 4 3 2 1

Highly descriptive Somewhat Not at all descriptive

1. **An IRB with members who are very knowledgeable about IRB procedures and legal requirements**

7 6 5 4 3 2 1

Essential Moderately Important Not important

I don’t know/
I have no experience

7 6 5 4 3 2 1

Highly descriptive Somewhat Not at all descriptive

1. **An IRB that reviews protocols in a timely fashion**

7 6 5 4 3 2 1

Essential Moderately Important Not important

I don’t know/
I have no experience

7 6 5 4 3 2 1

Highly descriptive Somewhat Not at all descriptive

1. **An IRB whose members do not allow personal biases to affect their evaluation of protocols**

7 6 5 4 3 2 1

Essential Moderately Important Not important

I don’t know/
I have no experience

7 6 5 4 3 2 1

Highly descriptive Somewhat Not at all descriptive

1. **An IRB that applies appropriately flexible standards regarding voluntary and informed consent requirements (e.g. required wording is less demanding for minimal risk research using competent adult participants)**

7 6 5 4 3 2 1

Essential Moderately Important Not important

I don’t know/
I have no experience

7 6 5 4 3 2 1

Highly descriptive Somewhat Not at all descriptive

1. **An IRB that recognizes when it lacks sufficient expertise to evaluate a protocol and seeks an outside evaluator**

7 6 5 4 3 2 1

Essential Moderately Important Not important

I don’t know/
I have no experience

7 6 5 4 3 2 1

Highly descriptive Somewhat Not at all descriptive

1. **An IRB that shows considerable evidence that the advancement of science is a part of its mission**

7 6 5 4 3 2 1

Essential Moderately Important Not important

I don’t know/
I have no experience

7 6 5 4 3 2 1

Highly descriptive Somewhat Not at all descriptive

1. **An IRB that is willing to work with investigators to find mutually satisfying solutions whenever disagreements exist**

7 6 5 4 3 2 1

Essential Moderately Important Not important

I don’t know/
I have no experience

7 6 5 4 3 2 1

Highly descriptive Somewhat Not at all descriptive

1. **An IRB that offers editorial suggestions regarding consent documents and protocols (e.g. typos, grammar, clarity)**

7 6 5 4 3 2 1

Essential Moderately Important Not important

I don’t know/
I have no experience

7 6 5 4 3 2 1

Highly descriptive Somewhat Not at all descriptive

1. **An IRB that provides a comprehensive training program for its new members**

7 6 5 4 3 2 1

Essential Moderately Important Not important

I don’t know/
I have no experience

7 6 5 4 3 2 1

Highly descriptive Somewhat Not at all descriptive

1. **An IRB that treats investigators with respect**

7 6 5 4 3 2 1

Essential Moderately Important Not important

I don’t know/
I have no experience

7 6 5 4 3 2 1

Highly descriptive Somewhat Not at all descriptive

1. **An IRB that conducts a conscientious and complete review of protocols**

7 6 5 4 3 2 1

Essential Moderately Important Not important

I don’t know/
I have no experience

7 6 5 4 3 2 1

Highly descriptive Somewhat Not at all descriptive

1. **An IRB that maintains complete and accurate records**

7 6 5 4 3 2 1

Essential Moderately Important Not important

I don’t know/
I have no experience

7 6 5 4 3 2 1

Highly descriptive Somewhat Not at all descriptive

1. **An IRB that is open to innovative approaches to conducting research**

7 6 5 4 3 2 1

Essential Moderately Important Not important

I don’t know/
I have no experience

7 6 5 4 3 2 1

Highly descriptive Somewhat Not at all descriptive

1. **An IRB that takes timely action when an investigator has violated the specifications of its rulings**

7 6 5 4 3 2 1

Essential Moderately Important Not important

I don’t know/
I have no experience

7 6 5 4 3 2 1

Highly descriptive Somewhat Not at all descriptive

1. **An IRB that is composed primarily of highly competent investigators**

7 6 5 4 3 2 1

Essential Moderately Important Not important

I don’t know/
I have no experience

7 6 5 4 3 2 1

Highly descriptive Somewhat Not at all descriptive

1. **An IRB that ensures that at least one member is knowledgeable about the content domain and discipline of submitted protocols**

7 6 5 4 3 2 1

Essential Moderately Important Not important

I don’t know/
I have no experience

7 6 5 4 3 2 1

Highly descriptive Somewhat Not at all descriptive

1. **An IRB that takes timely and appropriate action whenever scientific misconduct is alleged**

7 6 5 4 3 2 1

Essential Moderately Important Not important

I don’t know/
I have no experience

7 6 5 4 3 2 1

Highly descriptive Somewhat Not at all descriptive

1. **An IRB that views protection of human participants as its primary function**

7 6 5 4 3 2 1

Essential Moderately Important Not important

I don’t know/
I have no experience

7 6 5 4 3 2 1

Highly descriptive Somewhat Not at all descriptive

1. **An IRB that includes a complete rationale when it denies or mandates changes in a protocol based on criteria that are more stringent than or different from relevant laws or national guidelines**

7 6 5 4 3 2 1

Essential Moderately Important Not important

I don’t know/
I have no experience

7 6 5 4 3 2 1

Highly descriptive Somewhat Not at all descriptive

1. **An IRB that requires members to abstain from evaluating protocols whenever a real or apparent conflict-of-interest arises**

7 6 5 4 3 2 1

Essential Moderately Important Not important

I don’t know/
I have no experience

7 6 5 4 3 2 1

Highly descriptive Somewhat Not at all descriptive

1. **An IRB that is allocated sufficient resources to carry out functions efficiently and thoroughly**

7 6 5 4 3 2 1

Essential Moderately Important Not important

I don’t know/
I have no experience

7 6 5 4 3 2 1

Highly descriptive Somewhat Not at all descriptive

1. **An IRB that conducts a conscientious, informed analysis of potential benefits weighed against potential risks before making decisions**

7 6 5 4 3 2 1

Essential Moderately Important Not important

I don’t know/
I have no experience

7 6 5 4 3 2 1

Highly descriptive Somewhat Not at all descriptive

1. **An IRB that holds no preconceived biases against particular research techniques**

7 6 5 4 3 2 1

Essential Moderately Important Not important

I don’t know/
I have no experience

7 6 5 4 3 2 1

Highly descriptive Somewhat Not at all descriptive

1. **An IRB that offers investigators information to improve the chances of gaining IRB approval**

7 6 5 4 3 2 1

Essential Moderately Important Not important

I don’t know/
I have no experience

7 6 5 4 3 2 1

Highly descriptive Somewhat Not at all descriptive

1. **An IRB that does not use its power to suppress research that is otherwise methodologically sound and in compliance with relevant laws whenever it perceives potential criticism from outside the scientific community**

7 6 5 4 3 2 1

Essential Moderately Important Not important

I don’t know/
I have no experience

7 6 5 4 3 2 1

Highly descriptive Somewhat Not at all descriptive

1. **An IRB that gives a complete explanation for any required changes to or disapprovals of protocols**

7 6 5 4 3 2 1

Essential Moderately Important Not important

I don’t know/
I have no experience

7 6 5 4 3 2 1

Highly descriptive Somewhat Not at all descriptive

1. **An IRB that invites investigators to present their position whenever a question or concern about a research protocol arises**

7 6 5 4 3 2 1

Essential Moderately Important Not important

I don’t know/
I have no experience

7 6 5 4 3 2 1

Highly descriptive Somewhat Not at all descriptive

1. **An IRB that offers consultation during the development of research protocols or grant applications**

7 6 5 4 3 2 1

Essential Moderately Important Not important

I don’t know/
I have no experience

7 6 5 4 3 2 1

Highly descriptive Somewhat Not at all descriptive

1. **An IRB that offers investigators opportunities to be educated about relevant laws and national guidelines**

7 6 5 4 3 2 1

Essential Moderately Important Not important

I don’t know/
I have no experience

7 6 5 4 3 2 1

Highly descriptive Somewhat Not at all descriptive

1. **An IRB that responds in a timely manner to investigators’ inquiries about its processes and decisions**

7 6 5 4 3 2 1

Essential Moderately Important Not important

I don’t know/
I have no experience

7 6 5 4 3 2 1

Highly descriptive Somewhat Not at all descriptive

1. **An IRB that acknowledges full responsibility for its errors or delays in processing protocols and attempts to correct them as expeditiously as possible**

7 6 5 4 3 2 1

Essential Moderately Important Not important

I don’t know/
I have no experience

7 6 5 4 3 2 1

Highly descriptive Somewhat Not at all descriptive

1. **An IRB that is open and pleasant in its interactions with investigators**

7 6 5 4 3 2 1

Essential Moderately Important Not important

I don’t know/
I have no experience

7 6 5 4 3 2 1

Highly descriptive Somewhat Not at all descriptive

1. **An IRB whose Secretariat (or staff member in charge of IRB functions) has a background in conducting research**

7 6 5 4 3 2 1

Essential Moderately Important Not important

I don’t know/
I have no experience

7 6 5 4 3 2 1

Highly descriptive Somewhat Not at all descriptive

1. **An IRB that monitors the progress of each approved research project in line with relevant laws and national guidelines**

7 6 5 4 3 2 1

Essential Moderately Important Not important

I don’t know/
I have no experience

7 6 5 4 3 2 1

Highly descriptive Somewhat Not at all descriptive

1. **An IRB that requires its Chair be an experienced investigator**

7 6 5 4 3 2 1

Essential Moderately Important Not important

I don’t know/
I have no experience

7 6 5 4 3 2 1

Highly descriptive Somewhat Not at all descriptive

1. **An IRB that has a diverse membership (i.e., includes women, minorities and both junior and senior members of the institution)**

7 6 5 4 3 2 1

Essential Moderately Important Not important

I don’t know/
I have no experience

7 6 5 4 3 2 1

Highly descriptive Somewhat Not at all descriptive

1. **An IRB whose members fully understand and act within the scope of their function**

7 6 5 4 3 2 1

Essential Moderately Important Not important

I don’t know/
I have no experience

7 6 5 4 3 2 1

Highly descriptive Somewhat Not at all descriptive

1. **An IRB that is composed of more than one lay person**

7 6 5 4 3 2 1

Essential Moderately Important Not important

I don’t know/
I have no experience

7 6 5 4 3 2 1

Highly descriptive Somewhat Not at all descriptive

1. **An IRB that views its role as being an investigator’s ally rather than as being a hurdle to clear**

7 6 5 4 3 2 1

Essential Moderately Important Not important

I don’t know/
I have no experience

7 6 5 4 3 2 1

Highly descriptive Somewhat Not at all descriptive

1. **An IRB that does a good job of upholding participants’ rights while, at the same time, facilitating the conduct of research**

7 6 5 4 3 2 1

Essential Moderately Important Not important

I don’t know/
I have no experience

7 6 5 4 3 2 1

Highly descriptive Somewhat Not at all descriptive

1. **An IRB that is empathetic with the difficulties that can present themselves during the design or conduct of the research**

7 6 5 4 3 2 1

Essential Moderately Important Not important

I don’t know/
I have no experience

7 6 5 4 3 2 1

Highly descriptive Somewhat Not at all descriptive

1. **An IRB that holds no preconceived biases against particular research topics**

7 6 5 4 3 2 1

Essential Moderately Important Not important

I don’t know/
I have no experience

7 6 5 4 3 2 1

Highly descriptive Somewhat Not at all descriptive

1. **An IRB that can competently distinguish exempt from nonexempt research**

7 6 5 4 3 2 1

Essential Moderately Important Not important

I don’t know/
I have no experience

7 6 5 4 3 2 1

Highly descriptive Somewhat Not at all descriptive

1. **An IRB composed of members who arrive at meetings well-prepared.**

7 6 5 4 3 2 1

Essential Moderately Important Not important

I don’t know/
I have no experience

7 6 5 4 3 2 1

Highly descriptive Somewhat Not at all descriptive

1. **Demographic data**

(1) What research institution are you affiliated with?

Tick box list of 38 institutions

(2) Are you a current or former member of a local IRB?

Radio button: Yes-No

(3) Which of these broad types of human subject research have you been involved in the past 12 months?

Tick box list [multiple answers possible]:

- Research involving only data (whether anonymized or not)
- Research involving only secondary use of previously collected human tissue (whether anonymized or not)
- Interventional research regulated by the Health Sciences Authority (HSA) (e.g. clinical trials)
- Interventional research that is not regulated by HSA
- Research that is not covered by any of the above categories

(4) How many years have you been involved in biomedical or clinical research?

Radio button: 1-3, 4-6, 7-10, 11 plus

(5) Have you ever been involved in the submission of a research protocol to an IRB/HREC/REC in a foreign jurisdiction?

Radio button: Yes-No

Tick box: USA, UK, Australia, Canada, China, India, Continental Europe, ASEAN country, Other
