# Supplementary material for: Perspectives of Singaporean biomedical researchers and research support staff on actual and ideal IRB review functions and characteristics: A quantitative analysis
Source: PLoS One. 2020 Dec 31;15(12):e0241783. doi: 10.1371/journal.pone.0241783 (PMC7774925; doi:10.1371/journal.pone.0241783)
Supplement: S1 Table — (DOCX) [file pone.0241783.s002.docx]

**S1 Table:** Research Institutions that have notified the Ministry Of Health, Singapore, of their operations (as at 4 September 2018).

| Agency for Integrated Care Pte. Ltd. |
| --- |
| Agency for Science, Technology and Research (A*Star)* |
| Ang Mo Kio Thye Hua Kwan Hospital |
| Centre for Research on Rehabilitation and Protection |
| Centre International de Development Pharmaceutique Pte. Ltd. |
| Concord International Hospital |
| Covance (Asia) Pte. Ltd. |
| DotBio |
| Dover Park Hospice |
| DSO National Laboratories* |
| HCA Hospice Care |
| Health Promotion Board (HPB) |
| Health Sciences Authority (HSA)* |
| Invitrocue Pte. Ltd. |
| K2 Medical Clinic Pte. Ltd. |
| Lilly-NUS Centre for Clinical Pharmacology Pte. Ltd.* |
| Lucence Diagnostics Pte Ltd |
| Nanyang Polytechnic |
| Nanyang Technological University (NTU)* |
| National Environment Agency |
| National Healthcare Group Pte. Ltd. (NHG)* |
| National University Health System (NUHS)* |
| National University of Singapore (NUS)* |
| National Youth Sports Institute |
| Ngee Ann Polytechnic* |
| Raffles Hospital |
| Renci Hospital |
| Republic Polytechnic* |
| Singapore Armed Forces* |
| Singapore Health Services Pte. Ltd. (SingHealth)* |
| Singapore Institute of Technology* |
| Singapore Medical Innovative Technology Pte Ltd |
| Singapore Polytechnic* |
| Singapore University of Technology & Design (SUTD) |
| Sivantos Pte. Ltd.* |
| Sport Singapore* |
| St Luke’s Hospital |
| Temasek Polytechnic |

*Note.* * denotes that at least 1 respondent was affiliated with the research institution.
