# Supplementary material for: Perspectives of Singaporean biomedical researchers and research support staff on actual and ideal IRB review functions and characteristics: A quantitative analysis
Source: PLoS One. 2020 Dec 31;15(12):e0241783. doi: 10.1371/journal.pone.0241783 (PMC7774925; doi:10.1371/journal.pone.0241783)
Supplement: S3 Table — (DOCX) [file pone.0241783.s004.docx]

**S3 Table.** Comparison between our paired difference of ideal and actual scores with the difference of ideal and actual scores in other studies .

| **Item No.** | **Items** | **Chenneville et al. (2014) (Ideal - Actual) mean** | **Reeser et al. (2008) (Ideal-Actual) mean** | **Our paired difference (Ideal-Actual) mean**  **(SD)** |
| --- | --- | --- | --- | --- |
| 3 | An IRB that reviews protocols in a timely fashion | 1.67** | 0.98** | 2.83  (2.11) |
| 40 | An IRB that views its role as being an investigator’s ally rather than as being a hurdle to clear | 1.63** | 1.07** | 2.62  (2.39) |
| 22 | An IRB that is allocated sufficient resources to carry out functions efficiently and thoroughly | 1.95 | 0.44** | 2.51  (2.23) |
| 32 | An IRB that acknowledges full responsibility for its errors or delays in processing protocols and attempts to correct them as expeditiously as possible | 1.94 | 0.93** | 2.39  (2.20) |
| 5 | An IRB that applies appropriately flexible standards regarding voluntary and informed consent requirements (e.g., required wording is less demanding for minimal risk research using competent adult participants) | 0.73** | 0.65** | 2.36  (2.18) |
| 1 | An IRB that is open to reversing its earlier decisions (i.e., willing to carefully listen to investigators’ appeals) | 0.96** | 0.71** | 2.33  (1.84) |
| 14 | An IRB that is open to innovative approaches to conducting research | 1.41** | 0.89** | 2.32  (1.96) |
| 42 | An IRB that is empathetic with the difficulties that can present themselves during the design or conduct of the research | 1.34** | 0.53** | 2.32  (2.22) |
| 8 | An IRB that is willing to work with investigators to find mutually satisfying solutions whenever disagreements exist | 1.40** | 0.89** | 2.31  (1.96) |
| 31 | An IRB that responds in a timely manner to investigators’ inquiries about its processes and decisions | 1.80 | 0.68** | 2.24  (2.03) |
| 7 | An IRB that shows considerable evidence that the advancement of science is part of its mission | 1.39* | 0.46** | 2.07  (2.10) |
| 41 | An IRB that does a good job of upholding participants’ rights while, at the same time, facilitating the conduct of research | 1.48* | 0.43** | 2.06  (1.90) |
| 29 | An IRB that offers consultation during the development of research protocols or grant applications | 1.74 | 0.32** | 2.00  (1.96) |
| 20 | An IRB that includes a complete rationale when it denies or mandates changes in a protocol based on criteria that are more stringent than or different from relevant laws or national guidelines | 1.58 | 0.77** | 1.92  (2.17) |
| 25 | An IRB that offers investigators information to improve the chances of gaining IRB approval | 2.13 | 0.74** | 1.79  (1.93) |
| 30 | An IRB that offers investigators opportunities to be educated about relevant laws and national guidelines | 1.71 | -0.01** | 1.79  (2.19) |
| 23 | An IRB that conducts a conscientious, informed analysis of potential benefits weighed against potential risks before making decisions | 1.21* | 0.43** | 1.78  (1.76) |
| 6 | An IRB that recognizes when it lacks sufficient expertise to evaluate a protocol and seeks an outside evaluator | 1.32 | 0.57** | 1.77  (1.98) |
| 34 | An IRB whose Secretariat (or staff member in charge of IRB functions) has a background in conducting research | 0.93* | 0.72** | 1.73  (2.16) |
| 2 | An IRB with members who are very knowledgeable about IRB procedures and legal requirements | 1.62 | 0.43** | 1.70  (1.56) |
| 27 | An IRB that gives a complete explanation for any required changes to or disapprovals of protocols | 1.68 | 0.86** | 1.66  (1.69) |
| 17 | An IRB that ensures that at least one member is knowledgeable about the content domain and discipline of submitted protocols | 0.73** | 0.42** | 1.62  (1.91) |
| 28 | An IRB that invites investigators to present their position whenever a question or concern about a research protocol arises | 1.13* | 0.56** | 1.58  (1.79) |
| 44 | An IRB that can competently distinguish exempt from non-exempt research | 1.09* | 0.31** | 1.57  (1.83) |
| 43 | An IRB that holds no preconceived biases against particular research topics | 1.12 | 0.47** | 1.54  (1.81) |
| 26 | An IRB that does not use its power to suppress research that is otherwise methodologically sound and in compliance with relevant laws whenever it perceives potential criticism from outside the scientific community | 1.37 | 0.56** | 1.47  (1.75) |
| 24 | An IRB that holds no preconceived biases against particular research techniques | 1.21 | 0.39** | 1.46  (1.89) |
| 38 | An IRB whose members fully understand and act within the scope of their function | 1.29 | 0.79* | 1.41  (1.56) |
| 33 | An IRB that is open and pleasant in its interactions with investigators | 1.42 | 0.54** | 1.38  (2.03) |
| 10 | An IRB that provides a comprehensive training program for its new members | 2.10* | 0.63* | 1.36  (1.75) |
| 4 | An IRB whose members do not allow personal biases to affect their evaluation of protocols | 1.62 | 1.00 | 1.36  (1.49) |
| 16 | An IRB that is composed primarily of highly competent investigators | 1.64 | 0.12** | 1.21  (1.93) |
| 37 | An IRB that has a diverse membership (i.e., includes women, minorities and both junior and senior members of the institution) | 1.05 | -0.41** | 1.19  (1.83) |
| 11 | An IRB that treats investigators with respect | 1.81** | 0.46* | 1.09  (1.69) |
| 12 | An IRB that conducts a conscientious and complete review of protocols | 1.45** | 0.41* | 0.87  (1.36) |
| 35 | An IRB that monitors the progress of each approved research project in line with relevant laws and national guidelines | 1.68** | -0.05** | 0.82  (1.70) |
| 36 | An IRB that requires its Chair be an experienced investigator | 1.15 | -0.16** | 0.78  (1.74) |
| 18 | An IRB that takes timely and appropriate action whenever scientific misconduct is alleged | 1.30* | 0.34 | 0.73  (1.41) |
| 45 | An IRB composed of members who arrive at meetings well-prepared | 1.51** | 0.62 | 0.67  (1.30) |
| 19 | An IRB that views protection of human participants as its primary function | 0.63 | 0.07** | 0.66  (1.17) |
| 13 | An IRB that maintains complete and accurate records | 1.21** | 0.39 | 0.59  (1.31) |
| 15 | An IRB that takes timely action when an investigator has violated the specifications of its rulings | 1.51** | 0.27 | 0.57  (1.43) |
| 21 | An IRB that requires members to abstain from evaluating protocols whenever a real or apparent conflict-of-interest arises | 0.85* | 0.33 | 0.50  (0.85) |
| 9 | An IRB that offers editorial suggestions regarding consent documents and protocols (e.g., typos,  grammar, clarity) | 1.59** | -0.07 | 0.40  (2.12) |
| 39 | An IRB that is composed of more than one lay person | 0.27 | -0.80* | 0.20  (2.09) |

*Note*. * *p* < 0.05, ** *p* < 0.001 for 2 tailed tests. The items are arranged in descending order based on the mean of the paired difference. The difference between ideal and actual scores presented in this table and the difference between ideal and actual scores presented in Figure 1 and Table 5 are not the same. This is because results in this table reflect the pairwise difference, and additional missing data arises in the actual ratings from the fact that respondents could choose the “I don’t know/I have no experience” response to questions on how the actual IRB functions.
