# Supplementary material for: Perspectives of Singaporean biomedical researchers and research support staff on actual and ideal IRB review functions and characteristics: A quantitative analysis
Source: PLoS One. 2020 Dec 31;15(12):e0241783. doi: 10.1371/journal.pone.0241783 (PMC7774925; doi:10.1371/journal.pone.0241783)
Supplement: S4 Table — (DOCX) [file pone.0241783.s005.docx]

**S4 Table.** The 5 most important ideal IRB characteristics.

| **5 most important ideal IRB characteristics (rank: 1-5)** | | | | |
| --- | --- | --- | --- | --- |
| **Rank** | **Singapore sample** | **USNV sample: Keith-Spiegel et al. (2006)** | |  |
| 1 | An IRB that reviews protocols in a timely fashion (item 3) | An IRB that reviews protocols in a timely fashion (item 3) | |  |
| 2 | An IRB that does a good job of upholding participants’ rights while, at the same time, facilitating the conduct of research (item 41) | An IRB whose members do not allow personal biases to affect their evaluation of protocols (item 4) | |  |
| 3 | An IRB that is willing to work with investigators to find mutually satisfying solutions whenever disagreements exist (item 8) | An IRB that does a good job of upholding participants’ rights while, at the same time, facilitating the conduct of research (item 41) | |  |
| 4 | An IRB that responds in a timely manner to investigators’ inquiries about its processes and decisions (item 31) | An IRB that does not use its power to suppress research that is otherwise methodologically sound and in compliance with relevant laws whenever it perceives potential criticism from outside the scientific community (item 26) | |  |
| 5 | An IRB whose members do not allow personal biases to affect their evaluation of protocols (item 4) | | An IRB with members who are very knowledgeable about IRB procedures and legal requirements (item 2) | |
