# Supplementary material for: Perspectives of Singaporean biomedical researchers and research support staff on actual and ideal IRB review functions and characteristics: A quantitative analysis
Source: PLoS One. 2020 Dec 31;15(12):e0241783. doi: 10.1371/journal.pone.0241783 (PMC7774925; doi:10.1371/journal.pone.0241783)
Supplement: S5 Table — (DOCX) [file pone.0241783.s006.docx]

**S5 Table.** The 5 least important ideal IRB characteristics.

| **5 least important ideal IRB characteristics (rank: 41-45)** | | |
| --- | --- | --- |
| **Rank** | **Singapore sample** | **USNV sample: Keith-Spiegel et al. (2006)** |
| 41 | An IRB that has a diverse membership (i.e., includes women, minorities and both junior and senior members of the institution) (item 37) | An IRB that offers consultation during the development of research protocols or grant applications (item 29) |
| 42 | An IRB composed of members who arrive at meetings well-prepared (item 45) | An IRB that has a diverse membership (i.e., includes women, minorities and both junior and senior members of the institution) (item 37) |
| 43 | An IRB that is composed primarily of highly competent investigators (item 16) | An IRB that offers investigators opportunities to be educated about relevant laws and national guidelines (item 30) |
| 44 | An IRB that offers editorial suggestions regarding consent documents and protocols (e.g., typos, grammar, clarity) (item 9) | An IRB that offers editorial suggestions regarding consent documents and protocols (e.g., typos, grammar, clarity) (item 9) |
| 45 | An IRB that is composed of more than one lay person (item 39) | An IRB that is composed of more than one lay person (item 39) |
