# Supplementary material for: Perspectives of Singaporean biomedical researchers and research support staff on actual and ideal IRB review functions and characteristics: A quantitative analysis
Source: PLoS One. 2020 Dec 31;15(12):e0241783. doi: 10.1371/journal.pone.0241783 (PMC7774925; doi:10.1371/journal.pone.0241783)
Supplement: S7 Table — (DOCX) [file pone.0241783.s008.docx]

**S7 Table.** Comparison in expectation gap between Chenneville et al. (2014) study and our study.

| **Item No.** | **Item text** | **Study reporting a significantly smaller expectation gap*** |
| --- | --- | --- |
| 3 | An IRB that reviews protocols in a timely fashion | Chenneville et al. (2014) |
| 40 | An IRB that views its role as being an investigator’s ally rather than as being a hurdle to clear |  |
| 5 | An IRB that applies appropriately flexible standards regarding voluntary and informed consent requirements (e.g., required wording is less demanding for minimal risk research using competent adult participants) |  |
| 1 | An IRB that is open to reversing its earlier decisions (i.e., willing to carefully listen to investigators’ appeals) |  |
| 14 | An IRB that is open to innovative approaches to conducting research |  |
| 42 | An IRB that is empathetic with the difficulties that can present themselves during the design or conduct of the research |  |
| 8 | An IRB that is willing to work with investigators to find mutually satisfying solutions whenever disagreements exist |  |
| 7 | An IRB that shows considerable evidence that the advancement of science is part of its mission |  |
| 41 | An IRB that does a good job of upholding participants’ rights while, at the same time, facilitating the conduct of research |  |
| 23 | An IRB that conducts a conscientious, informed analysis of potential benefits weighed against potential risks before making decisions |  |
| 34 | An IRB whose Secretariat (or staff member in charge of IRB functions) has a background in conducting research |  |
| 17 | An IRB that ensures that at least one member is knowledgeable about the content domain and discipline of submitted protocols |  |
| 28 | An IRB that invites investigators to present their position whenever a question or concern about a research protocol arises |  |
| 44 | An IRB that can competently distinguish exempt from non-exempt research |  |
| 10 | An IRB that provides a comprehensive training program for its new members | Our study |
| 11 | An IRB that treats investigators with respect |  |
| 12 | An IRB that conducts a conscientious and complete review of protocols |  |
| 35 | An IRB that monitors the progress of each approved research project in line with relevant laws and national guidelines |  |
| 18 | An IRB that takes timely and appropriate action whenever scientific misconduct is alleged |  |
| 45 | An IRB composed of members who arrive at meetings well-prepared |  |
| 13 | An IRB that maintains complete and accurate records |  |
| 15 | An IRB that takes timely action when an investigator has violated the specifications of its rulings |  |
| 21 | An IRB that requires members to abstain from evaluating protocols whenever a real or apparent conflict-of-interest arises |  |
| 9 | An IRB that offers editorial suggestions regarding consent documents and protocols (e.g., typos, grammar, clarity) |  |

**Note*. The expectation gap is the (ideal-actual) score. A smaller expectation gap indicates that an IRB more closely met the respondents’ expectation. Comparison is performed at 95% confidence level.
