# Supplementary material for: Perspectives of Singaporean biomedical researchers and research support staff on actual and ideal IRB review functions and characteristics: A quantitative analysis
Source: PLoS One. 2020 Dec 31;15(12):e0241783. doi: 10.1371/journal.pone.0241783 (PMC7774925; doi:10.1371/journal.pone.0241783)
Supplement: S8 Table — (DOCX) [file pone.0241783.s009.docx]

**S8 Table**. Items where respondents with experience in data-only research score significantly differently from those without experience in data-only research.

| **Item No.** | **Item text** | **Ideal/ Actual** | **Respondents without experience in data-only research** | **Respondents with experience in data-only research** | **P-value** |
| --- | --- | --- | --- | --- | --- |
| 1 | An IRB that is open to reversing its earlier decisions (i.e., willing to carefully listen to investigators’ appeals) | Actual | 5  (5.75, 3.25) | 3.5  (4.75, 2) | 0.017 |
| 2 | An IRB with members who are very knowledgeable about IRB procedures and legal requirements | Actual | 5  (6, 5) | 4  (6, 4) | 0.007 |
| 5 | An IRB that applies appropriately flexible standards regarding voluntary and informed consent requirements (e.g., required wording is less demanding for minimal risk research using competent adult participants) | Actual | 5  (6, 3.5) | 3.5  (5, 1) | 0.017 |
| 7 | An IRB that shows considerable evidence that the advancement of science is part of its mission | Actual | 5  (6, 4) | 2  (4, 2) | 0.001 |
| 10 | An IRB that provides a comprehensive training program for its new members | Actual | 5  (6.5, 4.5) | 4  (5, 2) | 0.011 |
| 14 | An IRB that is open to innovative approaches to conducting research | Actual | 5  (5.5, 4) | 3  (5, 2) | 0.020 |
| 17 | An IRB that ensures that at least one member is knowledgeable about the content domain and discipline of submitted protocols | Actual | 6  (7, 4.25) | 4  (5, 3) | 0.014 |
| 20 | An IRB that includes a complete rationale when it denies or mandates changes in a protocol based on criteria that are more stringent than or different from relevant laws or national guidelines | Actual | 5  (6, 4) | 4  (6, 2.5) | 0.039 |
| 22 | An IRB that is allocated sufficient resources to carry out functions efficiently and thoroughly | Actual | 5  (6.75, 4) | 3  (4, 1) | p < 0.001 |
| 32 | An IRB that acknowledges full responsibility for its errors or delays in processing protocols and attempts to correct them as expeditiously as possible | Actual | 5  (6.25, 4) | 4  (6, 2) | 0.034 |
| 34 | An IRB whose Secretariat (or staff member in charge of IRB functions) has a background in conducting research | Actual | 6  (6, 4.25) | 4  (5, 2) | 0.008 |
| 40 | An IRB that views its role as being an investigator’s ally rather than as being a hurdle to clear | Ideal | 7  (7, 6) | 7  (7, 6) | 0.027 |
| 42 | An IRB that is empathetic with the difficulties that can present themselves during the design or conduct of the research | Ideal | 6  (7, 5) | 7  (7, 6) | 0.040 |
| 44 | An IRB that can competently distinguish exempt from non-exempt research | Actual | 6  (7, 4) | 5  (6, 3.25) | 0.044 |

*Note*. The median, third quartile and first quartile for each group are reported. The third and first quartiles are reported in parentheses. Quantiles are calculated using R’s default method.
