# Supplementary material for: Perspectives of Singaporean biomedical researchers and research support staff on actual and ideal IRB review functions and characteristics: A quantitative analysis
Source: PLoS One. 2020 Dec 31;15(12):e0241783. doi: 10.1371/journal.pone.0241783 (PMC7774925; doi:10.1371/journal.pone.0241783)
Supplement: S2 Fig — (DOCX) [file pone.0241783.s011.docx]

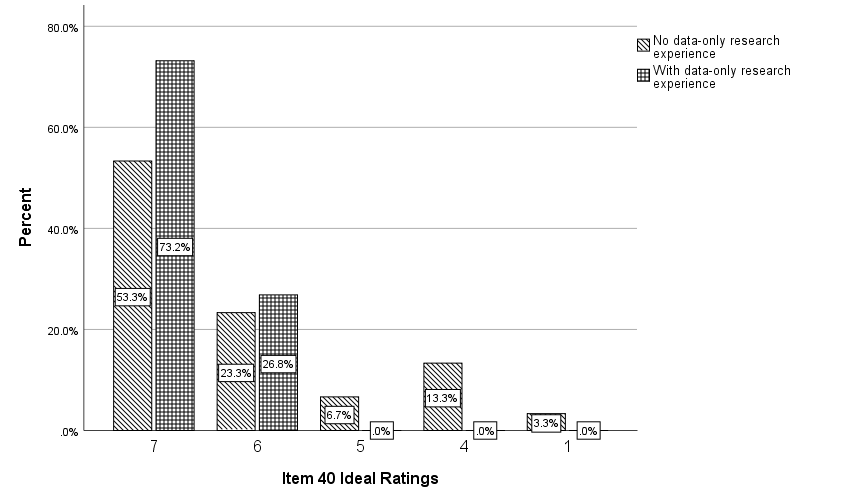


**S1 Figure**. Clustered bar chart showing the distribution of ideal ratings for item 40 for respondents with and without experience in data-only research.


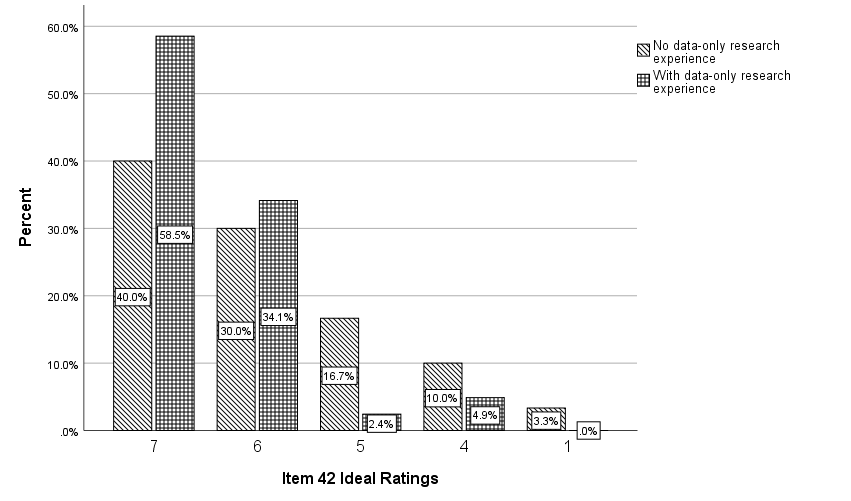


**S2 Figure**. Clustered bar chart showing the distribution of ideal ratings for item 42 for respondents with and without experience in data-only research.
